# Supplementary material for: The Molecular Mechanism of GhbHLH121 in Response to Iron Deficiency in Cotton Seedlings
Source: Plants (Basel). 2023 May 11;12(10):1955. doi: 10.3390/plants12101955 (PMC10224022; doi:10.3390/plants12101955)
Supplement: Supplementary file 1 [file plants-12-01955-s001.zip › Supplemental Figure.pdf]

Supplemental Figure

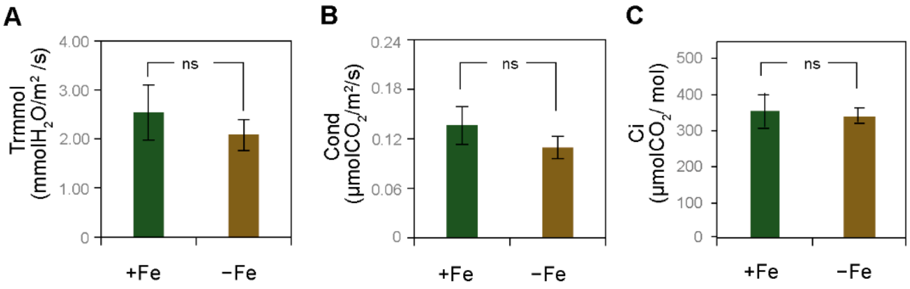

Supplemental Figure. S1 Iron deficiency reduces photosynthetic rate in the cotton leaves. Histograms of transpiration rate **(A)**, stomatal conductance **(B)**, and intercellular CO_2 concentration **(C)** in the cotton seedling leaves. Seedlings were grown on +Fe or –Fe solution. Trmmol: transpiration rate; Cond: stomatal conductance; Ci: intercellular CO_2 concentration. This Figure is supplementary data to **Figure 1 G**. Values represent means ± SD of three biological replicates. Significant differences were determined by Student's *t*-test, ns = no significance.

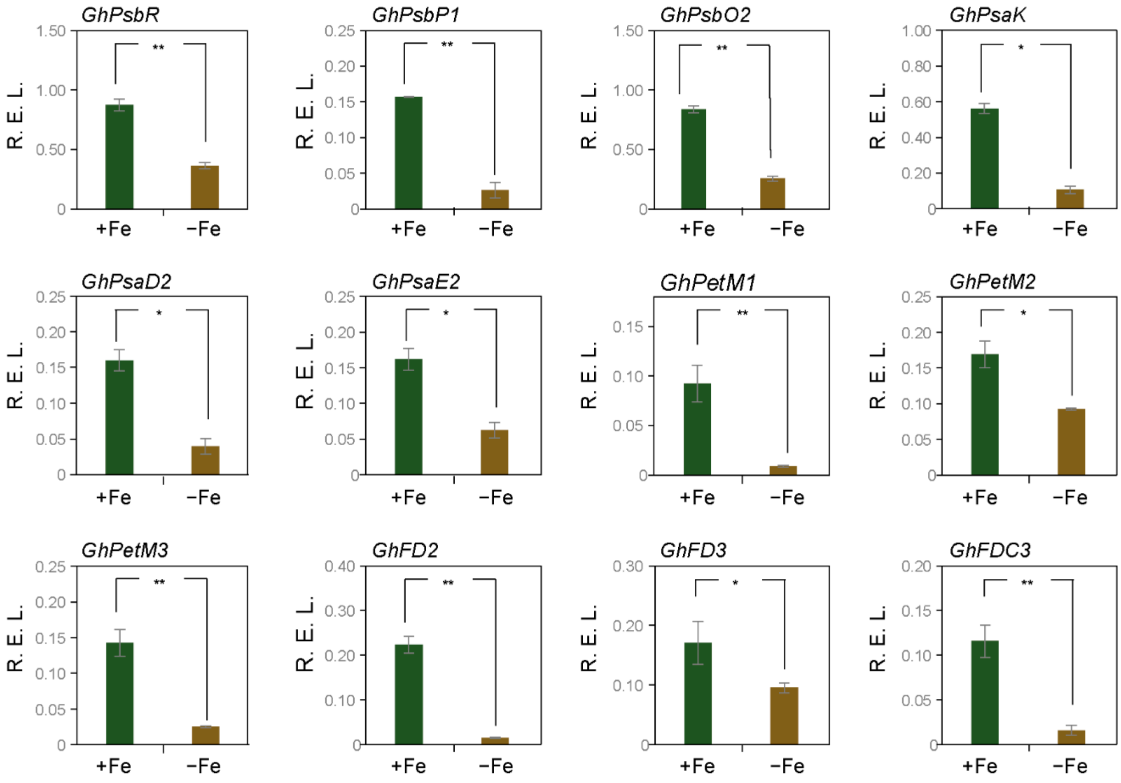

Supplemental Figure. S2 Iron deficiency reduces the expression of photosynthesis genes in cotton leaves.

Expression was determined by qPCR using RNA prepared from the leaves of cotton seedlings grown on +Fe or –Fe solution. Values represent means ± SD of three biological replicates. Significant differences were determined by Student's *t*-test, **P* < 0.05, ***P* < 0.01.

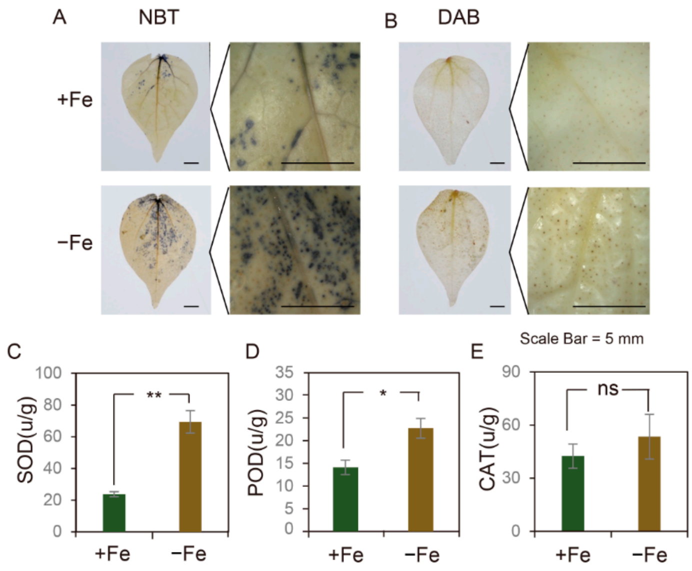

Supplemental Figure. S3 Iron deficiency affects the accumulation of reactive oxygen species (ROS) in cotton leaves. **(A, B)** Representative image showing NBT staining **(A)** and DAB staining **(B)** of cotton leaves. Seedlings were the same plants as in (**Figure1B**). Statistics were determined from three biological replicates, and each experiment contained ten seedlings. Scale bar = 5 cm. **(D-F)** Histograms of SOD activity **(D)**, POD activity **(E)**, and CAT activity **(F)** in cotton seedling leaves. Seedlings were the same plants as in (**Figure1C**). Values represent means ± SD of three biological replicates. Significant differences were determined by Student's *t*-test, **P* < 0.05, ***P* < 0.01, ns = no significance.

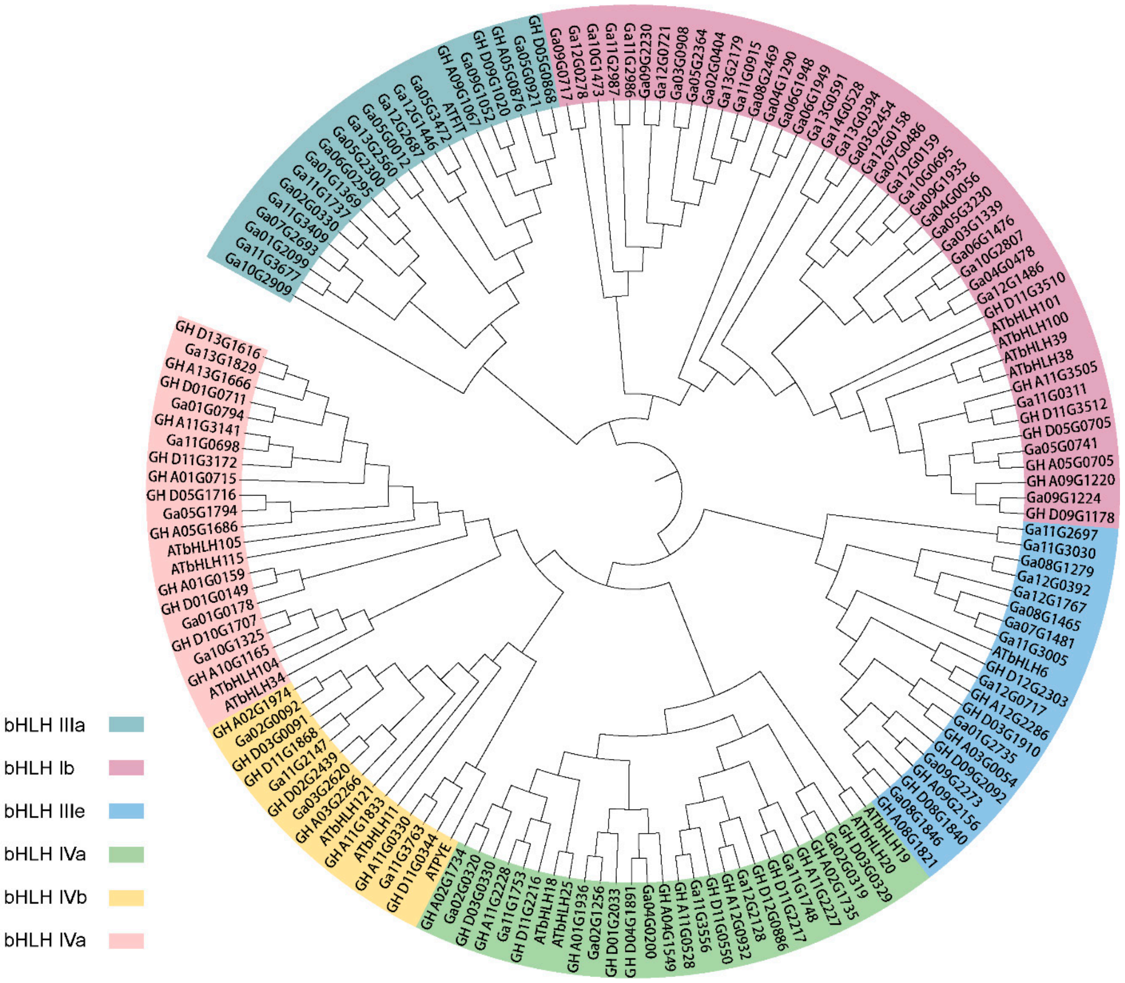

Supplemental Figure. S4 53 bHLH transcription factors involved in iron deficiency response in cotton. Phylogenetic tree of bHLH TFs involved in Fe homeostasis in *Arabidopsis thaliana* and cotton (*Gossypium hirsutum and Gossypium arboreum*). Protein sequences were retrieved from TAIR and COTTONOMICS. Sequences of bHLH transcription factors involved in Fe homeostasis were aligned using ClustalX 1.5. The phylogenetic tree was constructed by the neighbor-joining method using MEGA 3.1.

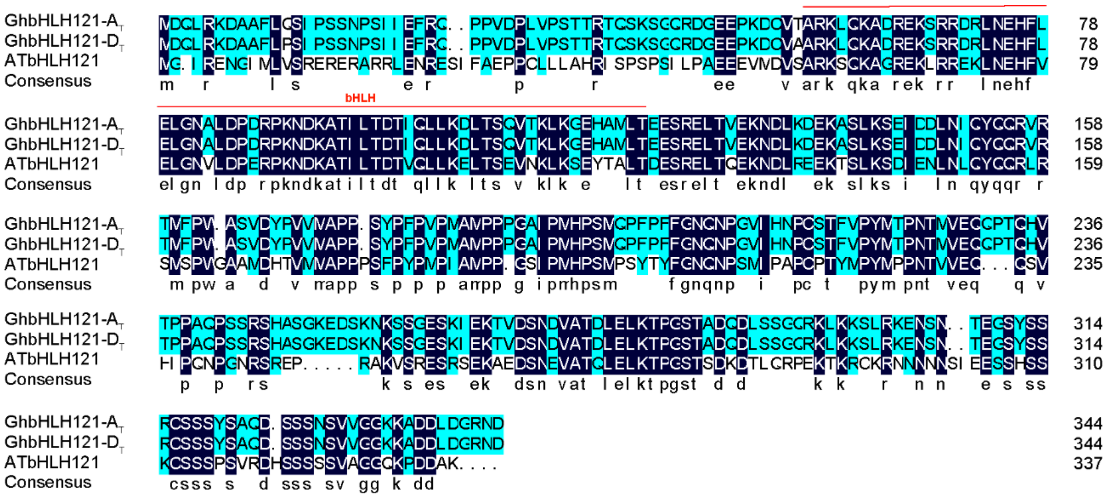

Supplemental Figure. S5 Alignment of the amino acid sequences of GhbHLH121-A_T, GhbHLH121-D_T, and AtbHLH121. Amino acid sequences were retrieved from TAIR and COTTONOMICS, and were aligned using DNAMAN. The red line represents the bHLH domain.

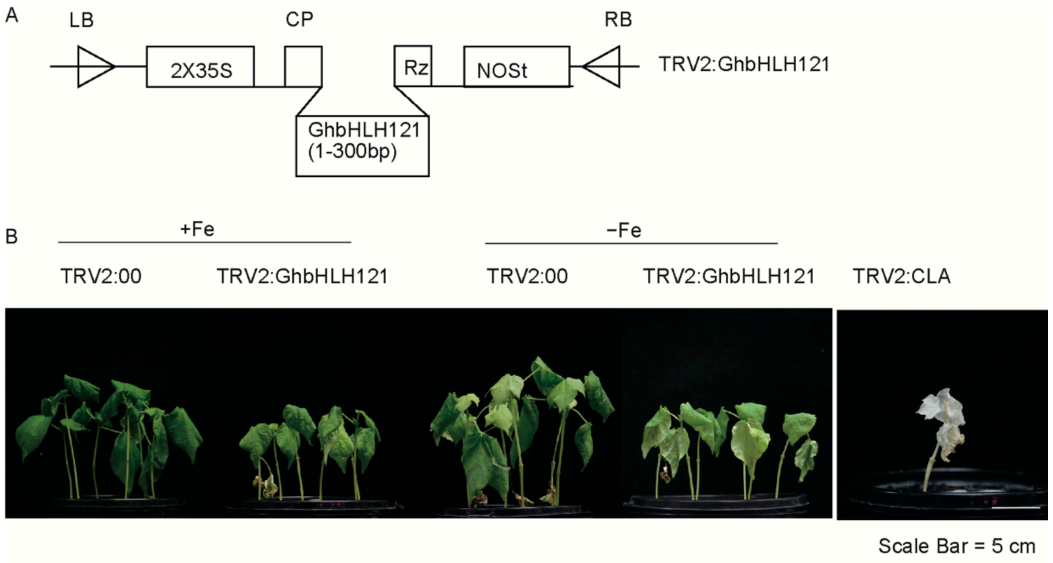

Supplemental Figure. S6 *TRV2:00* and the *TRV2:GhbHLH121* seedlings grown for three weeks on +Fe or –Fe solution. A: Construction of the *TRV2:GhbHLH121* VIGS vectors. B: Representative images showing the phenotypes of *TRV2:00* and the *TRV2:GhbHLH121* seedlings grown for three weeks on +Fe or –Fe solution. Statistics were determined from three biological replicates, and each experiment contained ten seedlings. Scale bar = 5 cm.

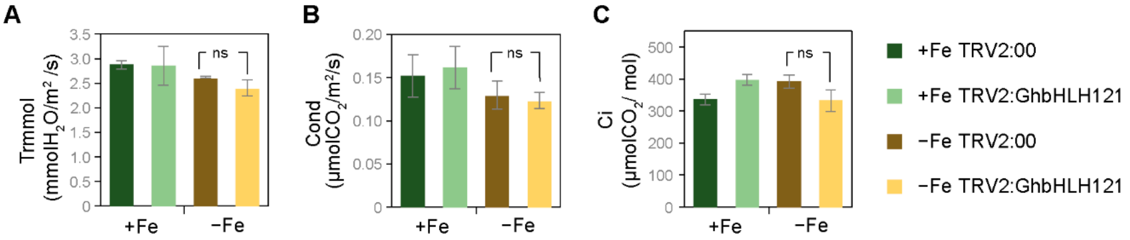

Supplemental Figure. S7 *TRV2:GhbHLH121* seedlings exhibit reduced photosynthetic rate under iron deficiency. Histograms of transpiration rate **(A)**, stomatal conductance **(B)**, and intercellular CO_2 concentration **(C)** in cotton leaves. *TRV2:00* and the *TRV2:GhbHLH121* seedlings were grown on +Fe or –Fe solution. Trmmol: transpiration rate; Cond: stomatal conductance; Ci: intercellular CO_2 concentration. Values represent means ± SD of three biological replicates. This Figure is supplementary data to (**Figure 3 H**). Significant differences were determined by Student's *t*-test, ns = no significance.

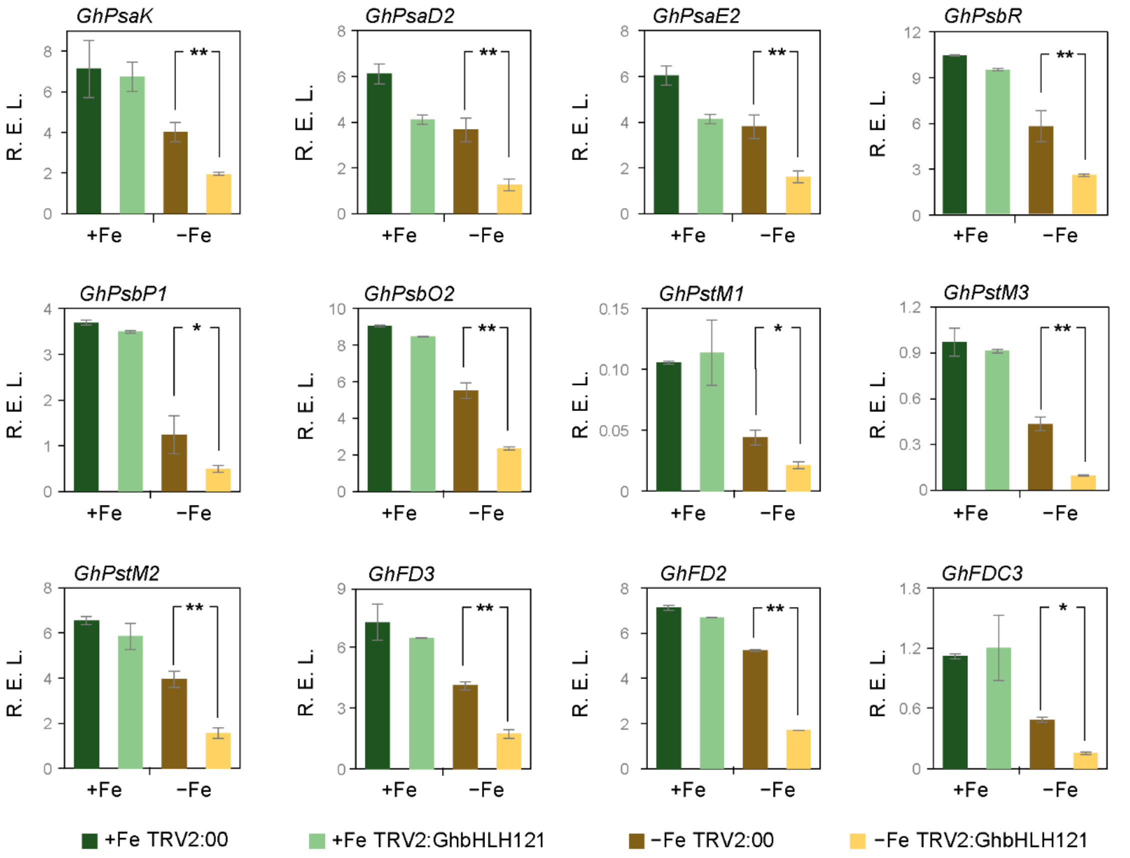

Supplemental Figure. S8 Suppression of *GhbHLH121* reduces the expression of photosynthesis genes under

iron deficiency. Expression was determined by qPCR using RNA prepared from the leaves of *TRV2:00* and *TRV2:GhbHLH121* cotton seedlings grown for three weeks on +Fe or –Fe solution. Values represent means ± SD of three biological replicates. Significant differences were determined by Student's *t*-test, $^{*}P < 0.05$, $^{**}P < 0.01$.

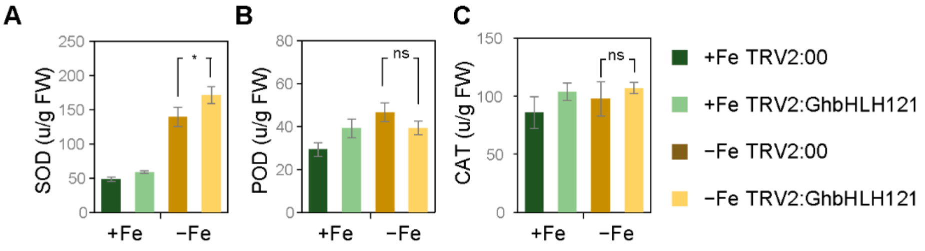

Supplemental Figure. S9 Suppression of *GhbHLH121* affects the accumulation of ROS under iron deficiency. Histograms of SOD activity **(A)**, POD activity **(B)**, and CAT activity **(C)** in cotton seedling leaves. Seedlings were the same plants as in (**Figure 3D**). Values represent means ± SD of three biological replicates. Significant differences were determined by Student's *t*-test, $^{*}P < 0.05$, ns = no significance.

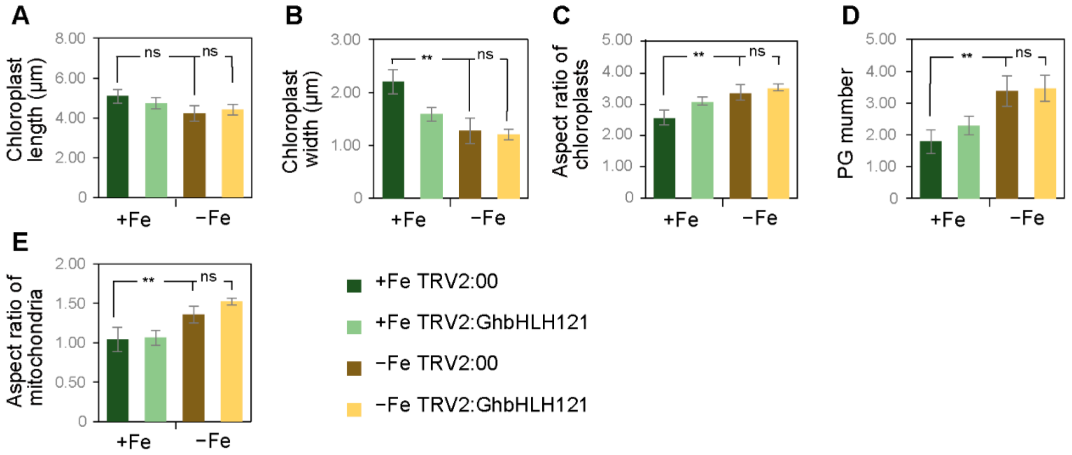

Supplemental Figure. S10 Iron deficiency causes changes in chloroplast shape. Histograms of the average length **(A)**, width **(B)**, aspect ratio **(C)**, and PG number **(D)** of chloroplasts and the aspect ratio of mitochondria **(E)** shown in **Figure 4**. Values represent means ± SD of three biological replicates. Significant differences were determined by Student's *t*-test, $^{**}P < 0.01$. ns = no significance.

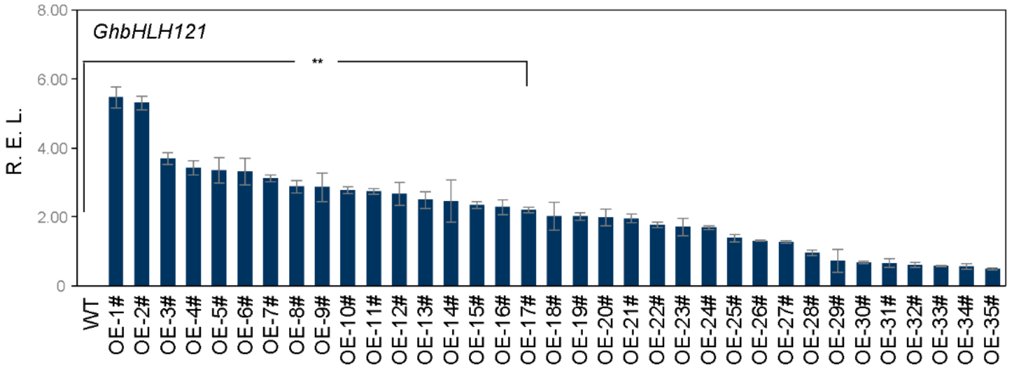

Supplemental Figure. S11 Expression of *GhbHLH121* in *GhbHLH121-OE* transgenic and wild-type *Arabidopsis*. Expression was determined by qPCR using RNA from seedlings grown for 7 days in $^1/_2$ MS medium. Values represent means ± SD of three biological replicates. Significant differences were determined by Student's *t*-test, **P* < 0.05, ***P* < 0.01.

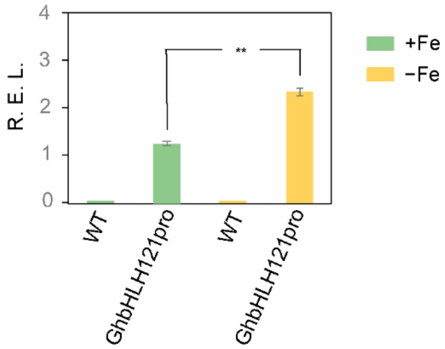

Supplemental Figure. S12 Histogram to show that GUS reporter expression in the WT and *Ghbhlh121pro::GUS Arabidopsis* seedlings grown for 1 weeks on +Fe or –Fe media. Values are means ± SD of three biological replicates. Significant differences were determined by Student's *t*-test, ***P* < 0.01.

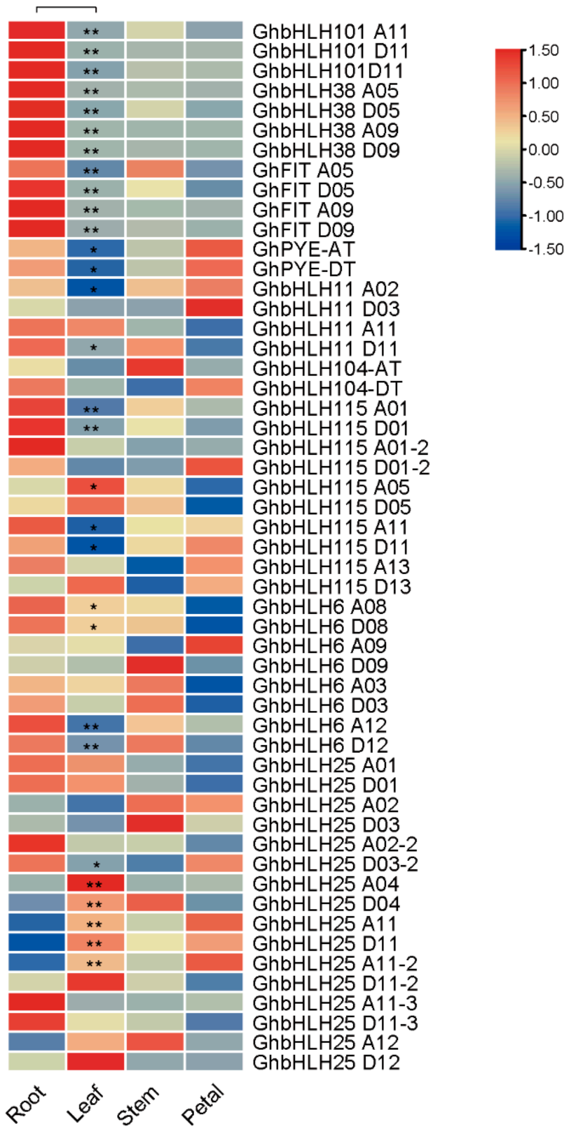

Supplemental Figure. S13 Expression of *GhbHLH* TFs involved in the iron deficiency response in cotton. Expression was determined by qPCR using RNA from cotton root, stem, leaf, and petal. Values represent means ± SD of three biological replicates. Significant differences were determined by Student's *t*-test, *$P < 0.05$, **$P < 0.01$.

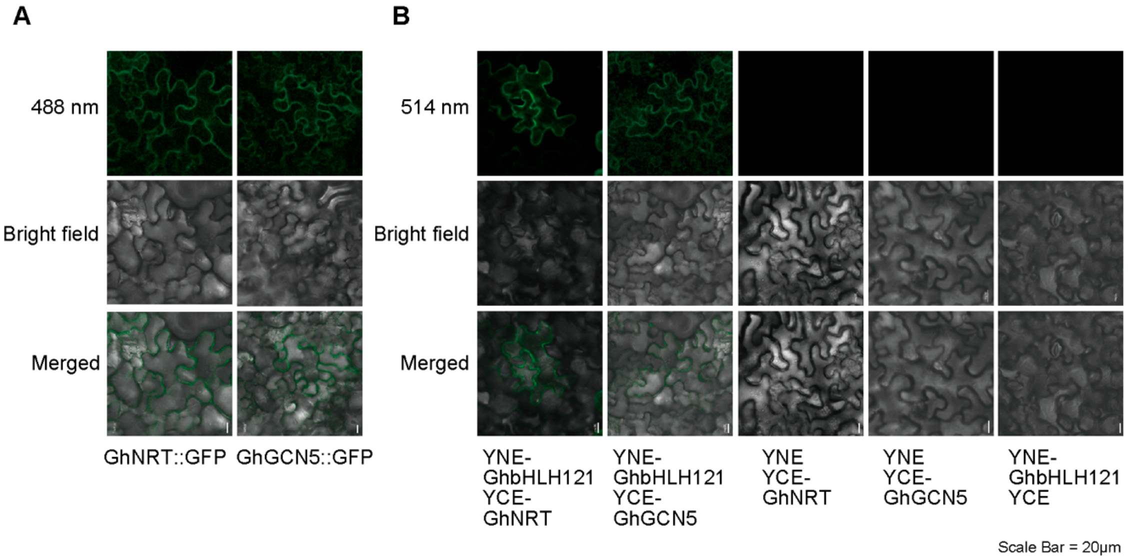

Supplemental Figure. S14 GhbHLH121 co-localizes with GhGCN5 and GhNRT in the cytoplasm. GhNRT and GhGCN5 were fused with the N-terminus of YFP (YNE) and GhbHLH121 with the C-terminus of YFP (YCE), then transferred into *N. benthamiana* leaves and visualized by confocal microscopy. Each experiment utilized three biological replicates. Scale bar = 20 µm.

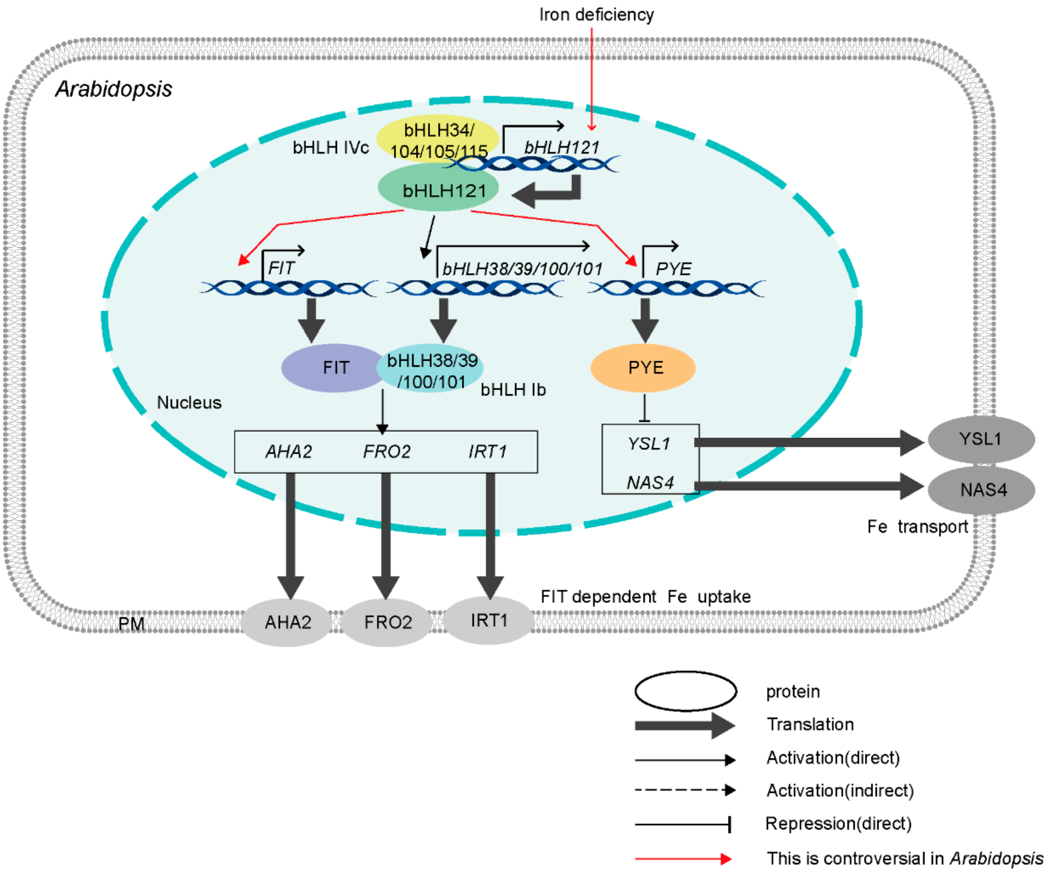

Supplemental Figure. S15 A working model illustrating the roles of AtbHLH121 in response to iron deficiency in *Arabidopsis*. When *Arabidopsis* subjected to iron deficiency, bHLH IVc promotes the transcription of *bHLH121*. bHLH121 recognizes the *FIT* promoter and functional interaction with bHLH IVc TFs to positively regulate *FIT* expression, which is controversial in *Arabidopsis*. FIT and bHLH Ib TFs then form heterodimers to promote the expression of Fe uptake genes, such as *IRT1* and *FRO2*. bHLH121 recognizes the *PYE* promoter and positively regulate *PYE* expression, which is controversial in *Arabidopsis*.
